# Sequence Heterogeneity of the ORF3 Gene of Porcine Epidemic Diarrhea Viruses Field Samples in Fujian, China, 2010–2012

**DOI:** 10.3390/v5102375

**Published:** 2013-09-30

**Authors:** Xi Chen, Lili Zeng, Jinxian Yang, Fusong Yu, Junqing Ge, Qing Guo, Xindang Gao, Tieying Song

**Affiliations:** 1 Biotechnology Institute, Fujian Academy of Agricultural Sciences, Fuzhou 350003, China; E-Mails: kobeid@163.com (X.C.); nks139@139.com (J.Y.); yufusong58@163.com (F.Y.); junqingg@163.com (J.G.); xindanggao@163.com (X.G.); 2 Fujian Hualong Group Feed CO., LTD, Fuzhou 350003, China; E-Mails: zenglili_2013@163.com (L.Z.); guoqinghl@126.com (Q.G.)

**Keywords:** porcine epidemic diarrhea virus, PEDV, heterogeneity, phylogenetic analysis, vaccine development

## Abstract

Twenty-seven field samples that showed positive in PEDV detection were collected from different farms of Fujian province from 2010 to 2012. Their heterogeneity was investigated by analysis of the ORF3 gene because of its potential function as a representation of virulence. According to the results, six Fujian strains in Group 1 showed a different genotype with unique point mutations, which might be used in differentiation between PEDV groups and brought potential antigenic variation. P55 and five reference strains in Group 2 had a long length deletion, showing another genotype and might be involved in the variation of virulence. Phylogenetic analysis revealed that the collected Fujian strains were very distant from the vaccine development strain CV777, which might be the reason why the vaccine was inefficient to control the disease. The results can help to reconsider the strategy of PEDV vaccine management and prevent outbreaks of PEDV-induced diarrhea more efficiently.

Porcine epidemic diarrhea virus (PEDV) causes severe entero-pathogenic diarrhea in piglets, especially in neonates, and the disease has a high mortality rate which can reach 80% in certain situations [1]. The disease was first recognized in England in 1971 [2], and since then, outbreaks of the disease were often reported in Europe and Asia [3,4,5,6]. Since the 1990s, a periodic vaccination strategy has been applied on pig farms nationwide to control the disease but these vaccines were not completely effective in preventing the disease, which led to a growing loss of newborn piglets in Fujian [7,8]. 

PEDV is an enveloped, single-stranded RNA virus belonging to the family *Coronaviridae* [9,10,11]. Its genome contains six ORFs, including pplab (pol), spike (S), membrane (M), ORF3, small membrane (sM), and nucleocapsid (N) [12,13]. The ORF3 encodes an ion channel protein and regulates virus production [14], and its loss might result in attenuation of the virus in the natural host [11]. The differentiation of ORF3 could be a marker of adaptation to cell culture and attenuation of virus [15], which could be a valuable tool to study the molecular epidemiology of PEDV [16]. The variation of field isolates of PEDV might change the genotypes and may be one of the possible reasons for the outbreaks in immunized pigs in Hebei, China [17]. Similar results were demonstrated in our primary study on field samples from 3 different swine farms in Fujian [18]. Thereby it is necessary to analyze the genetic heterogeneity of PEDV to find out which genotype prevails in Fujian. In this study, the ORF3 gene of PEDV field samples from different farms in Fujian province were cloned and sequenced for genetic diversity analysis.

Partial of intestine or stool specimens were taken individually from the acute enteritis and watery diarrhea of piglets from different big swine farms in the Fujian province between 2010 and 2012, and used for PEDV detection through PED Ag Test Kit (Bionote, Seogu-Dong, Korea). PEDV positive samples were used for sequence analysis and phylogenetic analysis. Intestinal samples were homogenized with 9 times of phosphate-buffered saline (PBS). The suspensions were then vortexed and centrifuged for 10 min at 1,700 × g. The supernatants were collected and stored at −80 °C before utilization.

Total RNA was extracted from the supernatants of the homogenized samples with the RNAiso Plus agent (Takara, Dalian, Japan) according to the manufacturer’s instructions. The forward and reverse primers [18], ORFS 5'-ACCGAGTTGAGACATACA-3' and ORFR 5'-GGAATAGAACCGTTAGACAT-3', were designed to amplify the ORF3 gene from the extracted RNA using Primescript^®^ One Step RT-PCR Kit Ver.2 (Takara, Dalian, Japan) under the following conditions: reverse transcription at 50 °C for 30 min, denaturation at 94 °C for 2 min, 30 cycles of denaturation at 94 °C for 30 s, annealing at 55 °C for 30 s and extension at 72 °C for 1 min. The RT-PCR products were analyzed by 1.5% agarose gel electrophoresis and visualized by ultraviolet illumination after ethidium bromide staining. Bands of the corresponding size of the gene were excised and the synthesized DNA was purified using QIAquick Gel Extraction Kit (QIAGEN, Dusseldorf, Germany) according to the manufacturer’s instructions, then sequenced by Takara Company.

The reference strains used for the sequence analysis were described in Table 1. Alignment and phylogenetic analysis of the nucleotide sequences of the ORF3 gene were performed with ClustalW method by Mega 4.0 program [19]. The antigenic indexes of the sequence were predicted using DNAMAN program. A phylogenetic tree was constructed with nucleotide and deduced amino acid sequences using the bootstrap neighbor-joining method separately [20]. The reliability of topologies was estimated by performing bootstrap analysis with 1,000 replicates. 

**Table 1:** Reference porcine epidemic diarrhea virus (PEDV) strains with 99% similarities for the ORF3 genes.

Reference strains or samples	Accession number	Source
attenuated DR13	JQ023162	South Korea
CV777 truncated ORF3	GU372744	Europe
CH/GSJIII/07	GU372743	Heilongjiang, North China
CH/BJ/2011	JQ027019	Heilongjiang, North China
DBI865 truncated ORF3 gene	HQ537432	South Korea
P55	JQ723734	Fujian, South China
Zhejiang-08	JX002703	Zhejiang, South China
DX	EU031893	South Korea
F422	JQ723733	Fujian, South China
GD-A	JX112709	Guangdong, South China
AJ1102	JX188454	Hubei, North China
P15	KC120789	Fujian, South China
P4	KC120784	Fujian, South China
P14	KC120779	Fujian, South China
P1	KC120802	Fujian, South China
P9	KC120787	Fujian, South China
M2227	HQ537439	South Korea
CH/S	JN547228	Heilongjiang, North China
CH/FJND-3/2011	JQ282909	Heilongjiang, North China
P37	KC120794	Fujian, South China
GD-B	JX088695	Guangdong, South China
P718	KC120798	Fujian, South China
P229	KC120799	Fujian, South China
P16	KC120790	Fujian, South China
P10	KC120782	Fujian, South China
P35	KC120793	Fujian, South China
P82	KC120796	Fujian, South China
P17	KC120791	Fujian, South China
BJ-2011-1	JX435306	Beijing, North China
P83	KC120797	Fujian, South China
P68	JQ723732	Fujian, South China
P81	KC120795	Fujian, South China
P32	KC120792	Fujian, South China
P6	KC120785	Fujian, South China
P2	KC120783	Fujian, South China
P84	KC120780	Fujian, South China
P19	KC120800	Fujian, South China
P18	KC120801	Fujian, South China
P7	KC120786	Fujian, South China
P85	KC120781	Fujian, South China
P13	KC120788	Fujian, South China
CV777	JN599150	Europe
LZC	EF185992	Gansu, North China

In this study, 27 samples were found to show positive results in PEDV detection. They were used to amplify the ORF3 gene of PEDV, and then the gene was cloned and sequenced for alignment and phylogenetic analysis. Nucleotide sequence analysis indicated that all the samples were separated into two groups (Figure 1). The ORF3 gene of all the samples, except P55, was 675 bp in length and encoded a protein of 224 amino acids, which was similar to 10 reference strains. Six of them (F422, P1, P4, P9, P14, and P15), together with three reference strains had eight unique point mutations and formed a subgroup in Group 1. However, only one local strain, P55, was 626 bp in length and encoded a protein of 92 amino acid, which was similar to five reference strains (CH/GSJIII/07, CV777 truncated ORF3, CH/BJ/2011 truncated ORF3, DBI865 truncated ORF3, Zhejiang-08) in Group 2. All the Group 2 strains had a significant deletion at nt 245–294, except that the attenuated DR13 strain had a similar deletion at nt 244–294.

In terms of predicted amino acid sequence, the Subgroup 1, including six local strains, had two unique mutations, from L to S at 25 and from C to F at 107 respectively (Figure 2). It also had a unique point mutation (from V to F at 80, Figure 2), which was similar to Group 2 strains (Figure 2). P55 was found to have a long length deletion from 82 aa to the end, which existed in most of the Group 2 strains (Figure 2). No difference was found in the other 20 Fujian samples when compared to the relevant reference strains. 

To analyze the phylogenetic relationships of the 27 Fujian samples and the reference strains from various parts of the world, we constructed a neighbor-joining phylogenetic tree using their ORF3 amino acid sequences (Figure 3). The results indicated that all the local stains were separated into three potential clusters, which might have different origins based on the topology. P55 was clustered into Group 2, while the others were clustered into Group 1. In Group 1, 6 Fujian samples clustered into one subgroup (Subgroup 1) and the other 20 samples were clustered into Subgroup 3. 

Although the bi-combined attenuated vaccine against TGEV and PEDV infection was authorized for utilization on swine farms in Fujian province, outbreaks of PED have caused tremendous economical losses [21]. It is necessary to characterize the genetic sequence of the PEDV field samples and find out how the prevalence happened. In this study, 27 PEDV Fujian samples were identified, whose ORF3 gene sequence analysis indicated that they could be separated into two general groups. P55 with five reference strains were clustered into Group 2, which had a long length deletion in both of the nucleotide and amino acid peptide sequences. It indicated that this genotype might have prevailed in China as four strains in this group were collected from China but from different provinces (Table 1). In addition, the virulence of the virus was reduced in cell culture adaptation through the deletion mutant [15], but those five strains with the region deletion were virulent field samples, which revealed that there might be other mutations related to the virulence.

Six Fujian strains, as well as three reference ones, were characterized by eight unique point mutations in the nucleotide sequence and three amino acid changes in the peptide. These were clustered into Subgroup 1 (Figure 1). It was noteworthy that these samples were collected from four different geography regions, including South Korea (DX), Hubei in North China (AJ1102), Guangdong in South China (GD-A), and Fujian in South China (F422, P1, P4, P9, P14, and P15), respectively. Whether any recombination occurred among the field strains in these areas needs further study, but those mutations might be used in differentiation between PEDV groups or new DNA markers in the PEDV field strains.

**Figure 1:**
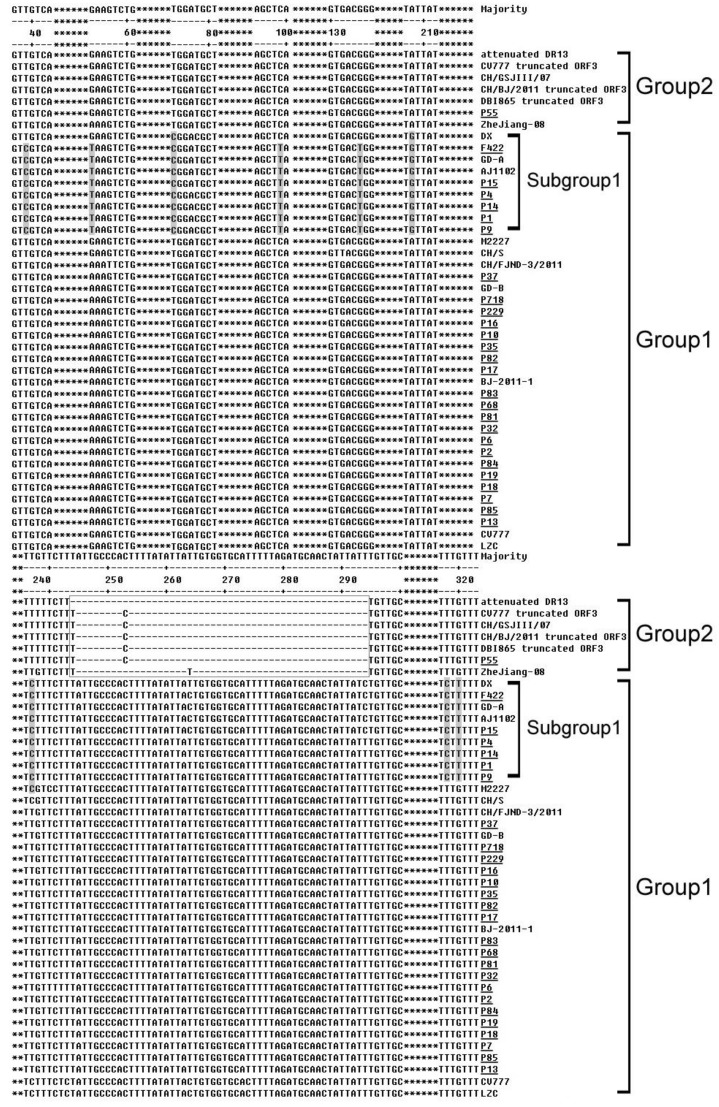
Alignment of nucleotide sequences of ORF3 genes of Fujian PEDV strains and reference strains. The asterisks represent the segments with no differences and not shown in this figure. The dashes represent deleted nucleotides. The shadows indicate the unique substitutions of chosen strains. The boxes indicate the unique insertions or deletions of chosen strains. The underlines represent the Fujian field samples in this study.

The ORF3 protein of reference strain CV777 had nine high antigenic indexes based on DNAMAN program analysis, which were located at amino acid 4–39, 41–64, 70–114, 118–124, 134–148, 158–174, 179–190, 192–207, and 211–221 plots, respectively. The strains in Subgroup 1 had amino acid changes at 25, 80 and 107 (Figure 2), located at the antigenic regions 4–39 and 70–114, respectively, which might alter their antigenicities and can be used as a label to remark this subgroup. No difference was found in the other regions.

**Figure 2:**
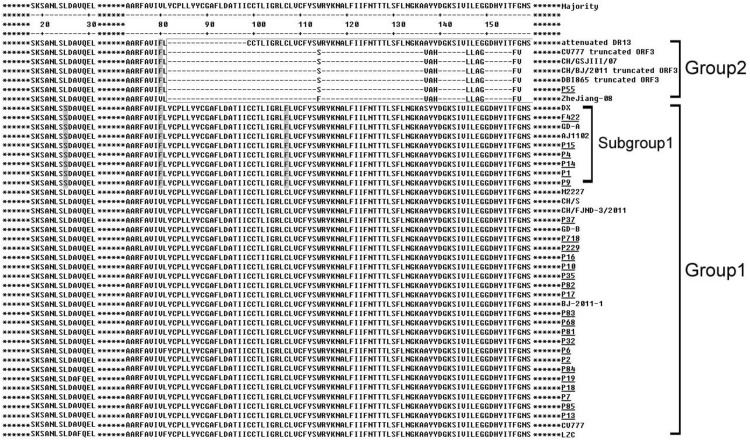
Alignment of amino acid sequences of ORF3 proteins of Fujian PEDV strains and reference strains. The asterisks represent the segments with no differences and not shown in this figure. The dashes represent deleted amino acids. The shadows indicate the unique substitutions of chosen strains. The boxes indicate the unique insertions or deletions of chosen strains. The underlines represent the Fujian field samples in this study.

Phylogenetic analysis indicated that all the strains were divided into 2 relevant groups (Group 1 and Group 2, Figure 3), and Group 1 was formed by three subgroups. None of the Fujian strains were observed to be close to vaccine development strain, CV777 [22]. That might explain why the vaccine was not efficient enough to prevent PED prevalence. In addition, as the samples were taken from farms that covered all the district of Fujian, the phylogenetic tree demonstrated that there might be three genotypes of PEDV prevailing in Fujian.

Similar results were achieved by Li *et al*. [23] although they investigated a different gene (S gene) in PEDV from nine farms in 2011. In their work, three new variants were identified from field diarrhea samples, which was similar to the status of Fujian local strains (F422, P1, P4, P9, P14, and P15) in Subgroup 1 (Figure 1, Figure 2 and Figure 3) in this study. The presence of another two field isolates, which shared high sequence identity with the attenuated strain DR13 from South Korean, was similar to P55 in the classification of Group 2 in this study (Figure 1 and Figure 2). Both of the results demonstrated that the effectiveness of the CV777-based vaccine was influenced and might lead to the outbreak of severe diarrhea on China’s pig farms.

**Figure 3:**
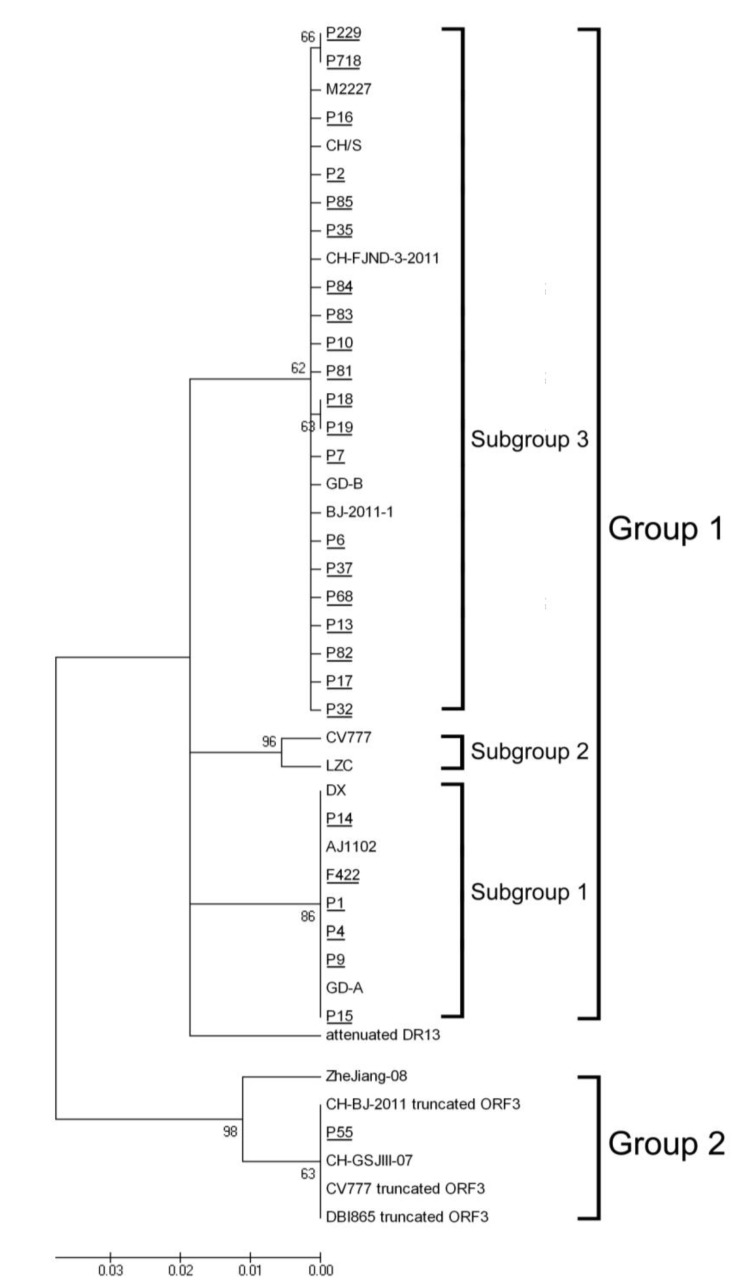
Phylogenetic tree of the nucleotide sequences of PEDV samples based on the ORF3 gene. Sequences of reference strains were obtained from the GeneBank database. GeneBank accession numbers were shown in Table 1. Tree topology was constructed using F84 model and bootstrap re-sampling (1,000 data sets) of the multiple alignments was used to test the statistical robustness of the trees obtained by NJ (using the program Neighbor from Mega v4.0 package) G1-G4: Divided groups by phylogenetic tree. The underlines represent the Fujian field samples in this study.

In conclusion, the PEDV field samples in Fujian province were characterized and compared by analyzing the sequence heterogeneity of ORF3 gene of PEDV. All the samples were separated into tw general groups, and the Fujian strain P55. In addition, five reference strains were clustered into Group 2, which had a long length deletion in both of the nucleotide and peptide sequences, showing a different genotype and might be involved in the variation of virulence. Six Fujian strains were clustered into Group 1, which showed another genotype with unique point mutations that might be used in differentiation between PEDV groups or new DNA markers and brought potential antigenic variation. Phylogenetic analysis revealed that the collected Fujian strains were very distant from the vaccine development strain CV777, which might be the reason why the vaccine was inefficient in controlling the disease. This study might help to choose an appropriate PEDV field strain as a vaccine candidate and efficiently prevent outbreaks of PED. 
